# Management of Cardiac Surgery in a Pandemic Region Hospital: Precautions, Results and Experiences During COVID-19

**DOI:** 10.5152/eurasianjmed.2021.20290

**Published:** 2021-10

**Authors:** Mehmet Isik, Serkan Yıldırım, Yüksel Dereli, Omer Tanyeli, Niyazi Görmüş

**Affiliations:** Department of Cardiovascular Surgery, Necmettin Erbakan University Meram School of Medicine, Konya, Turkey

**Keywords:** COVID-19, COVID-19 treatment, cardiovascular surgery, cardiopulmonary bypass, pandemic

## Abstract

**Objective:**

The aim of the present study was to share the experiences gained from emergency and semi-emergency cases of open heart surgery performed during the COVID-19 outbreak in Necmettin Erbakan University Meram Medical Faculty Hospital, which was defined as a pandemic hospital by Turkish Ministry of Health and provided third degree health services.

**Materials and Methods:**

A total of 54 patients were retrospectively analyzed between 23 March and 22 May 2020, who were diagnosed to have aortic dissection, coronary artery disease, and heart valve diseases.

**Results:**

Thirty-two CABG, 12 valve surgery, 6 aortic surgery, 4 CABG + valve surgeries were performed. During the postoperative follow-up of 11 patients, who were suspicious of COVID-19, 8 of them displayed respiratory problems and partial oxygen depletion and required continuous positive airway pressure. The hospitalization duration of COVID-19-suspicious patients were approximately 5 days longer than that of normal patients. In one of the patient, who was treated positive for COVID-19, acute coronary syndrome developed and CABG was performed following the treatment.

**Conclusion:**

During the pandemic period, acute cardiac diseases needing urgent surgery could be misdiagnosed because of similar symptoms with COVID-19 and the health care practitioners concentrated with the COVID-19 primarily. On the other hand, pandemic fear could cause delayed admission to the hospital and increased postoperative mortality and morbidity. When a COVID-19-positive or -suspicious patient undergo open-heart surgery, problems resulting from both COVID-19 infection and cardiopulmonary bypass–associated systemic effects could arise. The combination of these two cases could worsen the complications.

## Introduction

The outbreak of COVID-19, which started in China in December 2019, adversely affected the healthcare system of many countries. The first COVID-19 case of Turkey was detected on March 13th.^1^ At this point in time, many pandemic precautions carried out in other countries were also implemented in Turkey and in our clinic. One of the most important precautions was to postpone all elective surgeries, except for patients who needed emergency care. The aim was to reduce the pandemic burdens on our healthcare system, leave more resources for COVID-19 patients, and prevent the spread of the disease among regular patients during their hospital visits.

All healthcare professionals’ working conditions drastically changed during the pandemic. As expected, working conditions of cardiovascular surgery department also was changed, including flexible business hours depending on the workload, obligation to use personal protective equipment (PPE), and keeping of social distancing.

For cases to be taken to emergency cardiovascular operations under the COVID-19 pandemic, the issue of preoperative and postoperative management of patients, who have to be taken into emergency cardiovascular surgeries, will be made clear as new studies are published in the literature. Many unknown issues on this subject were experienced throughout the pandemic process. Moreover, the timing of the surgery in COVID-19-positive patients and the impact of it on mortality and morbidity of these patients were not clearly known.

Necmettin Erbakan University Meram Medical Faculty Hospital is defined as an institution providing a third degree healthcare, which was designated as a pandemic hospital by Turkish Ministry of Health. The aim of the present study was to share the experiences obtained from emergency and semi-emergency open-heart surgery cases performed during the COVID-19 outbreak.

## Materials and Methods

A total of 54 patients, who were diagnosed with aortic dissection, coronary artery disease, and heart valve diseases and underwent emergency and semi-emergency open heart surgery, were retrospectively analyzed between 23 March and 22 May 2020. Patients, who had vascular surgeries, endovascular procedures, and medical treatments, were excluded from the study. The patients’ information were obtained from the hospital software database and medical records as well as physician’s follow-up notes. The study was performed following the approval, from Necmettin Erbakan University Meram MedicineFaculty Ethical Committee (2020/2566). All patients provided their own written consent prior to the participation into the present study. Extra information pages were added into the patient information forms regarding the development of potential complications due to pandemic. The present study was conducted by the principles of the Helsinki Declaration.

In our hospital, patient services for COVID-19-positive, -suspicious, or -negative patients were placed in different floors in order to reduce the contact between them. Elevator entry and exits were also regulated based on the COVID-19 cases. The third floor of our hospital, where intensive care units of general surgery, chest diseases, and reanimation departments are located, were designed as intensive care units for COVID-19-positive or -suspicious cases. The intensive care unit of our clinic was independent from other COVID-19 intensive care units. It was located on the same floor (second floor) with other surgery rooms, and one door was directly opened to the surgery room.

At the beginning of the pandemic, our staffs, who work on patient services, intensive care units, and surgery rooms, were trained for the appropriate use of PPE, how to approach COVID-19-positive patients, and surgery room principles. PPEs were divided into two as normal and detailed. Surgical masks, eye goggles, bonnet, and gloves were provided as normal PPEs. For detailed PPEs, N95 masks, full-face masks, disposable laboratory coats, boots style long overshoes, and overalls were provided in addition to the normal PPEs. During the pandemic, all hospital workers were employed in the flexible shift system. Throughout the pandemic, pregnant staff were placed on leave and all out-of-town travels and nonobligatory time-offs were abolished. For all staff, daily fever measurements and regular virus screening tests were performed, even if they were non-symptomatic. The use of normal PPEs became mandatory for all healthcare staff in their workplace. Moreover, frequent hand washing and use of disinfectants were encouraged. The use of disposable medical products was sufficiently increased.

### Preoperative Preparation

All patients hospitalized for open-heart surgery were tested for COVID-19 (respiratory distress, fever, recent travels, and the area of residence). Suspected patients were consulted with infectious diseases department and tested for the virus (PCR). Patients with respiratory problems, especially those who came from quarantined areas, were isolated. The healthcare personnel, who were in charge with performing the follow-up and medical treatment of these patients, were provided with detailed PPE. Two-thirds of the patients, who underwent surgery, were diagnosed at our hospital’s emergency department and cardiology clinic. The rest of the cases were the patients who were examined and diagnosed in outer centers and sent to our clinic for surgery.

Based on the degree of urgency, the detailed preoperative preparation process of the patients was performed quickly. For all patients, preoperative hemogram, biochemistry, aPTT, INR, C-reactive protein (CRP), and chest x-ray images were examined. Preoperative virus test was performed on heart valve patients who had similar symptoms to COVID-19 (respiratory distress, pulmonary edema, or pneumonia findings). Computed thorax tomography was performed on some patients based on the recommendation of the department of infectious diseases. If the clinical status of the suspicious patients was stable, the surgeries were postponed until confirmations of negative test results taken 24 h apart were obtained. The patients, who had aortic dissection, ischemic ongoing coronary artery disease, and stuck valve, were operated immediately as they were considered positive without testing.

### Management of the Surgery Room

In our hospital, a small complex, which consists of three separate surgery rooms and is isolated from other surgery rooms, was determined for the general surgeries of COVID-19-suspect or -positive patients. Before the pandemic, there were three surgery rooms for cardiovascular surgery department. Two of them were actively used during the pandemic. The cardiovascular surgery rooms were equipped with high efficiency particulate air filters integrated laminar flow units.

The patients were routinely admitted to the surgical rooms with a surgical mask. If needed, nasal oxygen is provided under the mask. There was a separate anesthetic team specializing in cardiac surgery. The patients were anesthetized and followed up by this specialized team. Arterial and central venous catheterization procedures were performed with ultrasonography. Tracheal intubation was performed with a video laryngoscope to prevent any secretion exposure. Disposable anesthesia breathing circuit and aspiration set were used. All doors were kept closed during surgery in order to minimize air traffic.

### Postoperative Follow Up and Treatment

Cardiac surgery intensive care patient rooms were divided by glass walls; each was equipped with a ventilation system and had separate entrances with doors that can be closed if needed. Except for two isolation rooms, the other 7 rooms opened to the same corridor. During the pandemic, intensive care unit of our clinic was opened for postoperative patients of other departments with no known risk of COVID-19.

Each patient was assigned with a nurse for the postoperative follow-ups during their postoperative period. During the pandemic, the number and business hours of healthcare workers were regulated since cases were not accepted electively. Therefore, the traffic in intensive care was minimized.

COVID-19-positive or -suspected patients were placed in isolated rooms. Medical treatments were determined following consultation with the infectious diseases, chest diseases, and anesthesia departments. The healthcare staffs, who were responsible for these patients, were provided with detailed PPE. The other healthcare staff was provided with normal PPE.

Following the surgery, the intubated patients were transferred to the intensive care unit through a separate corridor opening to the intensive care unit of the cardiac surgery department. During the intubation procedure, normal PPE plus full face masks were used in order to minimize the risks of exposure to patient secretions. Intravenous antiemetic was performed approximately 30 minutes before extubation. Disposable breathing circuit, aspiration set, endotracheal tube, and bacteria filter were used in each patient. Oxygen were provided nasally to extubated patients under their surgical masks or provided only with reservoir masks, if necessary. During the pandemic, the process of patient exercises, such as walking at the intensive care corridor, were canceled. Mobilization procedures were limited only to the patient’s own room.

Disposable and transparent nylon covers with a size of 100 × 100 cm^2^ were used for CPR patients in order to prevent the spread of secretions while manual ventilation with bag valve mask. These covers were opened to cover the patient’s head and neck region and manual ventilation procedure was performed under the cover. When the patients left intensive care, the room and all medical devices were cleaned with chlorine-containing disinfectants.

After the pandemic, all of our clinic’s service rooms were converted into single patient rooms. During the service follow-ups, only one attended was accepted per patient. The attendants were questioned for recent travel to the risky areas and were selected from those without suspected COVID-19 infection. All other patient visits at the service and intensive care units were prohibited.

## Results

During the study period, a total of 54 open-heart surgeries were performed (36 men and 18 women). The number of the cases were reduced by approximately 2/3 compared with the same period of the previous year. Demographic characteristics of the patients are provided in Table 1.

Preoperative preparation of 16 patients was performed at the intensive care unit due to their health conditions. The rest of the patients were prepared at the cardiovascular service. Eight patients, who were operated for CAD, were attached with intra-aortic balloon pump. Three coronary artery patients were operated off-pump. Five patients had an excitus during the postoperative days 1–9. Two of them had CABG, one had Cabrol procedure, the other one underwent reoperation mitral valve replacement and tricuspid ring anuloplasty, and the last patient underwent mitral valve replacement and CABG. The cause of mortality was independent of an infection, except one of them. The CABG patient, who died from an infection, was attached to the postoperative breathing apparatus, and the cause of death was sepsis associated with pneumonia. The patient, who underwent Cabrol procedure with the diagnosis of aortic dissection, was operated under hypotensive shock conditions and died. It was learned that this patient had symptoms for a few days but was anxious due to a pandemic and, therefore, delayed admission to the hospital. It was determined that nine patients delayed their admittance to the hospital due to COVID-19 anxiousness. All of the detailed information about the surgery and diagnosis are provided in Table 2.

During the postoperative follow-ups, 8 of 11 patients, who were suspected with COVID-19, developed respiratory problems and partial oxygen depletion. No lung disease anamnesis was detected in patients’ background checks. These patients had continuous positive airway pressure, bronchodilator therapy, and breathing exercises. Dual antibiotic treatment for duration of 15–21 days was started to those with a high risk of infection following a consultation with the department of infectious diseases. Of these patients, *Staphylococcus haemolyticus* growth was observed in the blood culture of the patient who was operated upon for type 1 dissection. Likewise, *Streptococcus sanguinis* was reproduced in the blood culture of the patient who was operated with the diagnosis of infective endocarditis. In the patients who had respiratory problems, there were no growth in their blood cultures and their PCR test results were negative. COVID-19 suspected patients stayed in the hospital approximately 5 days longer than an average patient was.

An 80-year-old patient, who was admitted to our clinic with a diagnosis of severe aortic and mitral stenosis, developed respiratory distress, and elective intubation was performed on the patient while waiting for the PCR test results. Following 2 days, this patient had a high fever and the lung X-ray indicated a lobar pneumonia and diffuse infiltration. Deep tracheal aspiration was performed twice, and the PCR results were negative. However, the patient died before being operated.

One patient, who was diagnosed with COVID-19, had acute coronary syndrome developed during the treatment. A bypass decision was made as a result of coronary angiography. However, the PCR test results were negative, which was tested at the 12th and 13th day of the treatment. Since the patient was unstable, as soon as observing the regression of infection parameters 15 day following the start of COVID-19 treatment, surgery was performed upon consulting with the department of infectious diseases. The patient was discharged from the hospital at postoperative day 11.

A patient who had been operated upon for infective endocarditis in the native mitral valve was pre-diagnosed with COVID-19 and treatment was performed by the department of infectious diseases for 5 days. When a bacterial agent was produced in the patient’s blood cultures and endocarditis was suspected, the diagnosis was made by performing echocardiography.

## Discussion

Cancelling elective cases and performing the surgeries of only emergency cases during the pandemic period was an important step to reduce the spread of the virus. On the other hand, allocating all opportunities to patients with COVID-19 may cause problems in the diagnosis and treatment of some disease groups. Some cardiology and cardiovascular surgery clinics in our city cancelled cases except for emergency cases in order to organize the bed needs of patients with COVID-19. The extent to which this situation will affect the diagnosis and treatment of people at risk of cardiovascular disease in the society will emerge over time. The surgical burden of our clinic was reduced by approximately 65% during the pandemic due to reduction of outpatient services and the elimination of elective coronary angiography procedures. Unexpected emergency cases, such as acute aortic dissection, were operated upon immediately. Surgery was performed on patients with no suspected COVID-19 and who had left main, left main like, or who were diagnosed with three vascular diseases, and surgery had been decided. The questions about whether non-revascularized CAD will cause a new infarction or how long can revascularization could safely be postponed will remain unclear. Therefore, it is very difficult to determine the limits clearly when defining the emergency case in cardiovascular surgery.

It has been reported that the incubation period of COVID-19 is 4–6 days, and sometimes it can be extended up to 14 days.^2^ Patients are usually admitted to hospitals with mild symptoms, such as low fever, cough, headache, fatigue, or asymptomatically.^2^ The mild or asymptomatic course of the disease makes many emergency open-heart surgery patients COVID-19 suspicious. More care should be taken to these patients who showed similar symptoms to COVID-19, including the cases coming from quarantine areas as well as patients hospitalized for valve surgery with respiratory distress. If the general condition of this group of patients was sufficient enough, a virus test should be performed by consulting with the department of infectious diseases. For emergency cases, similar procedures and care should be applied and performed in COVID-19-positive patients. Moreover, false negative results that may result from insufficient sampling or poor test sensitivity pose a great risk to healthcare workers.

To protect healthcare workers, the use of PPE is vital. Procedures should also be developed especially for intubation, extubation, and tracheostomy where patients are exposed to aerosols.^3^ Although healthcare professionals at the forefront of the fight against COVID-19 focus on protecting the health of other people, they also need to be protected from the epidemic in order to provide long-term service. Getting used to PPE and producing services using equipment was a challenge at first. Considering the second wave of the pandemic, the use of PPE negatively affects surgical performance, and the necessity to design more useful equipment has been reported.^4^

In the early days of the pandemic, fear, anxiety, and loss of concentration were observed in the employees of our clinic during the examination or surgery of COVID-19-suspected patients. It was observed that anxiety disappeared and motivation increased again as the surgeries of suspicious patients were successfully performed and patients discharged from the hospital. With the spread of the pandemic in the society, 2 people from our clinic’s assistant health personnel also became positive for COVID-19. Interestingly, it was found that these people had COVID-19 by out-of-hospital contact. The probable reason to explain this situation may be increased attention to the use of PPE in the hospital. Our employees returned to their jobs with home isolation and outpatient care without the need for intensive care.

The new recognition of the COVID-19 clinical course in detail may increase problems for the disease diagnosis. In the present study, one problem was more pronounced, especially among valve patients. During the pandemic period, admission of patients usually into the emergency room and any delays in transthoracic or transesophageal echocardiography procedures may cause false diagnosis or late diagnosis. Since during this period the first diagnosis that comes to mind in most patients with symptoms similar to COVID-19 is COVID-19, there may be delays and difficulties in making the correct diagnoses. Moreover, the psychological pressure that the pandemic creates on patients and physicians and the fear for starting the treatment late could lead to false COVID-19 diagnosis and then, in turn, could case mistreatment.

Nine patients, who underwent surgery, stated that they had pandemic-associated anxiety coming to the hospital despite their complaints and postponed admittance for a few days. This suggests a positive correlation between pandemic-associated anxiety and postoperative mortality and morbidity. Prolonged ischemia can further impair ventricular function and cause mechanical complications of myocardial infarction. Delayed surgery may increase mortality in aortic dissection cases. Such delays could cause deaths even before the admittance of the patients to the hospitals.

It has been reported that 19% of COVID-19-positive patients had cardiac damage that further increased the mortality.^5^ These patients displayed elevated levels of cardiac injury biomarkers. Moreover, in these patients, cardiac failure, ischemia-induced myocardial damage, and stunned myocardium were reported.^6,7^ Laboratory results suggested an increased lymphopenia, thrombocytopenia, lactate dehydrogenase, CRP, D-dimer, fibrinogen, ferritin, and interleukin-6, as well as decreased antithrombin.^8-^^10^ Some hemostatic conditions, including disseminated intravascular coagulation, have been reported to occur more frequently in COVID-19-positive patients.^10–12^ In the current study, postoperative leukocyte, platelet, and CRP tests were routinely monitored. No apparent abnormal results were observed except for leukocyte and CRP elevation. A systemic inflammatory response might be responsible for early increase in CRP and leukocyte numbers. However, further large-scale studies are required to justify the impact of COVID-19 on postoperative laboratory results.

One theory to explain the impact of COVID-19 on cardiovascular system states that the imbalance between the increased metabolic demand and reduced cardiac reserves could lead to heart failure. It has also been reported that infection can trigger acute coronary syndrome by causing an increased inflammatory response and coagulation, thereby causing myocardial depression, heart failure, and arrhythmias.^13^ Various cytokines have been suggested in the pathogenesis of the disease.^6^ Some of these cytokines have been reported to induce vascular permeability and leakage, thereby leading to pulmonary edema, impaired air exchange, ARDS, acute cardiac injuries, and multiorgan failure.^14^ One of our CABG patient developed acute coronary syndrome while receiving COVID-19 treatment. This patient had diabetes and hypertension as a risk factor for coronary artery disease. The combination of risk factors and COVID-19 may have worsened the existing clinical situation of the patient.

The negative effects of extracorporeal circulation used in open-heart surgery on heart, lung, and immune response are well known. The constant contact of the blood with the nonendothelial surface stimulates the systemic inflammatory response. The inflammatory response can have different effects on many organs. It could cause ARDS, especially in the lungs. Since COVID-19 also causes similar effects in lungs, the combination of the inflammatory response and the viral infection together could significantly increase the likelihood of ARDS. Moreover, it has been shown that blood transfusion, performed at a considerable rate in cardiac surgery, also causes lung damage.^15^ Postoperative ARDS could cause prolonged intensive care stay as well as increase the mortality.

Apart from extracorporeal circulation during cardiac surgery, surgical trauma, anesthesia, cardioplegia, myocardial ischemia, cardiac manipulation, heparin, and protamine are conditions that can cause systemic inflammatory response.^16^ During cardiopulmonary bypass (CPB), high-dose heparin is required to circulate the blood outside the endothelium without clotting. COVID-19 infection has been reported to affect the coagulation system.^2^ In the present study, active coagulation time, platelet count, INR, aPTT, and PT were monitored more frequently during and after CPB in COVID-19-suspected cases. The patients were examined postoperatively for deep vein thrombosis. No extra anticoagulant or antiaggregant treatment was required outside of the routine.

Off-pump techniques, heat regulation, heparin-coated perfusion circuits, complement inhibitors, glucocorticoids, and modified ultrafiltration are recommended to reduce the inflammatory response in cardiopulmonary bypass.^17^ Other conditions that may affect the systemic inflammatory response are minimally invasive extracorporeal circulation and cell savers. Both have been reported to have positive effects on cell protection, immunity, and reducing systemic cytokine load.^18^

Although it has not been proven, it has been stated that Covid-19 transmission may occur due to pneumoperitoneum, during laparoscopic surgery, or other surgical procedures through aerosols caused by cautery.^19,20^ There is no pneumoperitoneum-like condition in open heart surgery, but cautery is used in many stages to minimize bleeding. Especially, in CABG surgeries, continuous cautery use is required when harvesting the internal mammary artery. At this stage, aerosols originating from cautery can spread from the thoracic cavity to the operation room. It may be helpful to avoid the use of cautery as much as possible.^21^

It is recommended to postpone elective or semielective heart surgeries until the COVID-19 test results are negative at least twice 24 hours apart.^2^ In the present study, the test results were waited upon for COVID-19-suspected semi-emergency patients. However, in emergency, cases such as aortic dissection, stuck valve, and severe LMCA stenosis, the patients were operated without waiting due to higher risk of mortality and morbidity. Patients with suspected COVID-19 acquired respiratory distress and long-term antibiotic therapy as well as required long-term intensive care during the postoperative follow-up period. Although six of these patients do not have any indication in their blood culture, viral infection or CPB could be responsible for the effects observed. The impact of CBP on postoperative virus test results as well as the effects of antibiotic treatment on negative virus test results should be investigated further.

Elective cardiac surgical interventions are not recommended for patients suffering from acute viral infections. In such patients, postoperative acute respiratory distress syndrome may develop leading to increased risk of mortality.^22^ In the current situation, a new definition of safe surgery should be made.^23^

Performing a heart surgery on COVID-19-positive or -suspicious patients could significantly increase virus or CBP-associated systemic problems. The combination of these two conditions could increase the severity of complications. Therefore, we believe that all these conditions should be taken into consideration when making the surgical decision. As the experiences of COVID-19-positive patients are shared all over the world, the precautions to be taken during surgery and postoperative care will become clear.0

**Main Points**

- During the pandemic period, the first diagnosis that comes to mind in patient diagnosis is COVID-19. Therefore, cardiac surgery patients, who displayed similar symptoms, could be misdiagnosed. Moreover, the psychological pressure on both physicians and patients as well as the fear for delaying the treatment could cause misdiagnosis of COVID-19 leading to the mistreatment.
- On the other hand, pandemic fear could cause delayed admission to the hospital and increased postoperative mortality and morbidity.
- When COVID-19 positive or suspicious patient undergo open-heart surgery, problems resulting from both COVID-19 infection and cardiopulmonary bypass associated systemic effects could arise. Combination of these two cases could worsen the complications.

## Figures and Tables

**Table 1.** Demographic Characteristics of Patients

Demographic Characteristics	Patient Number (n = 54)
Average age	58.4 (37–80)
Cigarette smoking	24
Hypertension	21
Diabetes mellitus	16
Cerebrovascular accident	4
Chronic lung diseases	4
Malignancy history	2
Chronic renal failure	1
Body mass index	27.8 (23–39.5)

**Table 2.** Performed Surgeries and Diagnostic Information

Patient Number (n = 54)	Diagnosis	Diagnosis Details	Performed Surgeries	Number of COVID-19 Suspicious Patients
32	CAD	LMCA, LMCAL, 3 vascular disease, left ventricle aneurysm	CABG on-pump/off-pump	3
5	Aortic dissection	Type 1 dissections	Ascending aorta/hemiarcus replacement + debranching, Bentall, Cabrol	1
4	Mitral valve disease	Stenosis, insufficiency	MVR	2
1	Aortic valve disease	Stenosis, insufficiency	AVR	
3	Double valve disease	Stenosis, insufficiency balloon valvuloplasty	MVR + TRA, AVR + MVR	2
1	Ascendant aortic aneurysm	Sinus valsalva aneurysm	Bentall	
1	Infective endocarditis	Vegetation on the cap	MVR	1
4	CAD + valve disease	Stenosis, insufficiency, Pannus in aortic valve	AVR + MVR **+ **CABG, AVR + CABG, MVR + CABG	1
3	Reoperations	Stuck valve, SVO	MRA + TRA, MVR + TRA, AVR	1

AVR: aortic valve replacement, CAD: coronary artery disease, CVA: cerebrovascular accident, LMCA: left main coronary artery, LMCAL: left main coronary artery-like, MRA: mitral ring anuloplasty, MVR: mitral valve replacement, TRA: tricuspid ring anuloplasty
